# ﻿Scratching the surface: a new species of Bent-toed gecko (Squamata, Gekkonidae, *Cyrtodactylus*) from Timor-Leste of the *darmandvillei* group marks the potential for future discoveries

**DOI:** 10.3897/zookeys.1139.96508

**Published:** 2023-01-11

**Authors:** Kin Onn Chan, L. Lee Grismer, Fernando Santana, Pedro Pinto, Frances W. Loke, Nathan Conaboy

**Affiliations:** 1 Lee Kong Chian Natural History Museum, National University of Singapore, 2 Conservatory Drive, 117377 Singapore, Singapore National University of Singapore Singapore Singapore; 2 Herpetology Laboratory, Department of Biology, La Sierra University, 4500 Riverwalk Parkway, Riverside, San Diego, California 92505, USA La Sierra University Riverside United States of America; 3 Department of Herpetology, San Diego Natural History Museum, PO Box 121390, San Diego, California, 92112, USA Department of Herpetology, San Diego Natural History Museum San Diego United States of America; 4 Department of Protected Areas and National Parks, Ministry of Agriculture and Fisheries, Dili, Timor-Leste Department of Protected Areas and National Parks, Ministry of Agriculture and Fisheries Dili Timor-Leste; 5 Conservation International Singapore, 42B Boat Quay, Singapore 049831, Singapore Conservation International Singapore Singapore Singapore; 6 Conservation International Timor-Leste, Rua Dom Aleixo Corte Real, Mandarin, Dili, P.O. BOX 006, Timor-Leste Conservation International Timor-Leste Dili Timor-Leste

**Keywords:** Biogeography, Gekkota, lizards, phylogenetics, systematics, taxonomy, Wallacea

## Abstract

A new species of limestone-dwelling Bent-toed gecko (genus *Cyrtodactylus*) is described from Nino Konis Santana National Park in the far-east region of Timor-Leste. Both genetic and morphological data strongly support the evolutionary distinctness of the new species, which we describe herein as *Cyrtodactylussantana***sp. nov.** Phylogenetic analysis based on the ND2 mitochondrial gene inferred the new species as part of the *C.darmandvillei* group with close genetic affinities to *C.batucolus*, *C.seribuatensis*, *C.petani*, *C.sadleiri*, and two undescribed lineages from the Moluccas in Indonesia. The new species represents the first species of *Cyrtodactylus* identified at the species level from Timor-Leste and fills an important gap in our understanding of the biogeography and evolutionary history of *Cyrtodactylus* especially in the Wallacean region. Our results strongly suggest that the diversity of *Cyrtodactylus* in Wallacea is still underestimated and many more unnamed species remain to be described.

## ﻿Introduction

**Figure 1.**
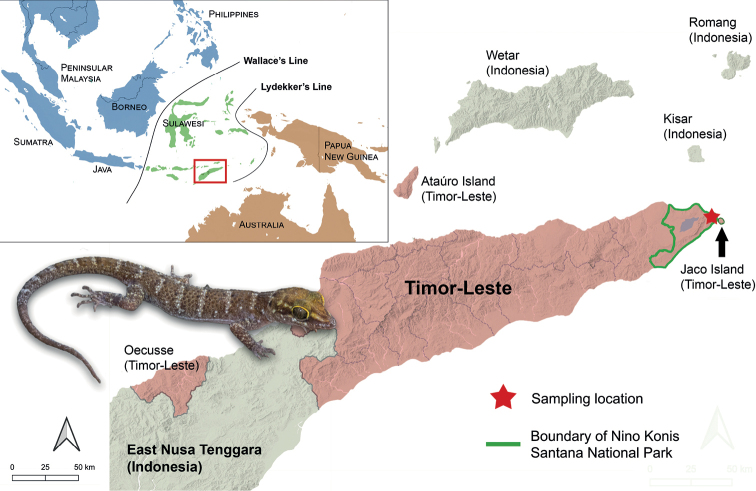
Upper left inset: Map of Sundaland (blue), Wallacea (green), and the Australo-Papuan region (orange) including Wallace’s and Lydekker’s lines that demarcates the boundaries of the three biogeographic regions. The red box denotes Timor and the surrounding islands. Right: An expanded map of Timor-Leste and the surrounding islands. Territories belonging to Timor-Leste are shaded in red. The red star indicates the location of the field site in the northeastern sector of the Nino Konis Santana National Park.

The Southeast Asian island of Timor is the largest of the Lesser Sunda Islands and is located within the biogeographical region of Wallacea, bounded by Wallace’s Line in the west and Lydekker’s Line in the east. The Democratic Republic of Timor-Leste (hereafter referred to as Timor-Leste) is a sovereign country occupying the eastern half of the island of Timor and includes the islands of Ataúro, Jaco, and the semi-enclave of Oecusse, a Special Administrative Region located in the western part of Timor. The western half of Timor is part of the Indonesian province of East Nusa Tenggara (Fig. 1). The terrain of Timor-Leste is mostly rugged and mountainous, with a central mountain range stretching east to west, reaching an elevation of 2,986 m at Mount Ramelau, the highest mountain on the island of Timor. The steep terrain slopes towards the north and the south, forming coastal versants dissected by alluvial outwashes, riverine plains, and wetland areas. Timor-Leste has a dry tropical climate with a pronounced dry season that lasts longer in the northern portion of the island, typically from May to November. Consequently, forest habitats are generally semi-deciduous and drought-adapted in the north and evergreen in the south. Other major habitat types include coral reefs, seagrass meadows, tropical montane forest, beach forest, coastal scrub, savannah woodland, open eucalyptus forest, swamps, mangroves, and a variety of agricultural lands such as coffee plantations and paddy fields. A more comprehensive review of Timor-Leste’s geography and environment can be obtained from Trainor et al. (2007) and Kaiser et al. (2011).

The herpetofaunal diversity of Timor-Leste is relatively poorly known, largely due to centuries of conflict and political instability that have hampered biological research. The first comprehensive report on the herpetofauna of Timor-Leste was published by Kaiser et al. (2011) followed by several complementary surveys throughout various parts of the country (Sanchez et al. 2012; Kaiser et al. 2013; O’Shea et al. 2015). These initial reports documented several undescribed species of *Cyrtodactylus* indicating that the diversity of this group of lizards in Timor-Leste is still underestimated and lack a formal scientific description. In August 2022, we surveyed the eastern region of Timor-Leste including Nino Konis Santana National Park (NKS) in the municipality of Lautém. Established in 2007, NKS is the first national park in Timor-Leste and encompasses the entire eastern tip of Timor, including Jaco Island (Fig. 1). It is mostly characterised by lowland tropical forest and includes several limestone caves that are of archaeological importance (García-Diez et al. 2020; O’Connor et al. 2021). It was during a survey of two of these caves that we discovered a population of *Cyrtodactylus* that is morphologically and genetically distinct from all other described congeners. In this study, we present evidence supporting the recognition of the *Cyrtodactylus* from NKS as a distinct evolutionary lineage followed by its description as a new species.

## ﻿Materials and methods

### ﻿Sampling and phylogenetic analysis

Fieldwork was conducted at the adjacent caves of Lene Hara and Napana Wei (8.411758°S, 127.293321°E; 152 m a.s.l.) in the northeastern sector of Nino Konis Santana National Park (**NKS**) on 30 August 2022. Specimens were euthanised using MS-222, fixed in 10% formalin, and transferred to 70% ethanol for long-term preservation. Liver samples were dissected and stored in 95% ethanol before fixation. All specimens are deposited at the Zoological Reference Collection (**ZRC**) of the Lee Kong Chian Natural History Museum, Singapore (**LKCNHM**).

We selected three (of the ten collected) specimens for DNA sequencing. The NADH dehydrogenase subunit 2 (ND2) mitochondrial gene was sequenced using the primers ﻿L4437 (AAGCTTTCGGGCCCATACC) and H5934 (AGRGTGCCAATGTCTTTGTGRTT) (Macey et al. 1997). The following PCR thermal protocol was used: initial denaturation at 95 °C for 5 min, followed by 35 cycles of a second denaturation at 94 °C for 60 s, annealing at 58 °C for 60 s, and cycle extension at 72 °C for 60 s. The newly generated sequences are accessioned at GenBank under the numbers OP650033–OP650035. An additional 350 sequences were obtained from GenBank representing six outgroup taxa and 344 ingroup taxa comprising all published ND2 sequences of described and undescribed *Cyrtodactylus* (Suppl. material 1: table S1). Sequences were assembled and aligned (MUSCLE algorithm) using Geneious v. 5.6.7 (Kearse et al. 2012). A partitioned maximum likelihood phylogenetic analysis was performed using IQTREE 2 (Minh et al. 2020). The sequence alignment was divided into four partitions comprising the 1^st^, 2^nd^, and 3^rd^ codon positions of the ND2 gene, and all tRNAs combined. The TEST function was implemented to determine the best-fit partition model using ModelFinder (Kalyaanamoorthy et al. 2017). Branch support was assessed via 1000 ultrafast bootstrap replicates (Hoang et al. 2017). Uncorrected *p*-distances were calculated using the complete deletion option in MEGA-X (Kumar et al. 2018). A Bayesian phylogeny was also inferred using BEAST2 v. 2.7.0 (Bouckaert et al. 2014) following the same partition scheme. Substitution models were averaged using the BEAST plugin bModelTest (Bouckaert and Drummond 2017). Two separate MCMC chains were executed (30,000,000 generations per chain) and subsequently assessed for convergence using Tracer v. 1.7 (Rambaut et al. 2018). Converged MCMC runs (ESS > 200) were combined and the first 10% of sampled trees were discarded as burn-in. The BEAST module TreeAnnotator was used to generate a Maximum Clade Credibility tree. The BEAST2 analysis was performed through the CIPRES Science Gateway portal (Miller et al. 2010).

### ﻿Morphology

The following morphological data were collected following Grismer et al. (2022): Snout-vent-length (**SVL**) = tip of snout to vent; axila-groin length (**AG**) = posterior margin of forelimb at its insertion point on the body to anterior margin of hind limb at its insertion point on the body; humeral length (**HumL**) = proximal end of humerus at its insertion point in the glenoid fossa to distal margin of elbow while flexed 90°; forearm length (**ForL**) = posterior margin of elbow while flexed 90° to inflection of the flexed wrist on the ventral side; femur length (**FemL**) = proximal end of femur at insertion point in the acetabulum to distal margin of knee while flexed 90°; tibial length (**TibL**) = posterior margin of knee while flexed 90° to base of heel on the ventral side; head length (**HL**) = posterior margin of retroarticular process of lower jaw to tip of snout; head width (**HW**) = distance across angle of jaws; head depth (**HD**) = maximum height of head from occiput to base of lower jaw posterior to eyes; eye diameter (**ED**) = greatest horizontal diameter of eye-ball; eye-to-ear distance (**EE**) = anterior edge of ear opening to posterior edge of the bony orbit; eye-to-snout distance (**ES**) = anteriormost margin of the bony orbit to tip of snout; eye-to-nostril distance (**EN**) = anterior margin of the bony orbit to posterior margin of the external nares; interorbital distance (**IO**) = distance between dorsomedial-most edges of the bony orbits; ear length (**EL**) = greatest oblique length across the auditory meatus; internarial distance (**IN**) = distance between the external nares across the rostrum; supralabials (**SL**) = largest scale at the corner of mouth or posterior to eye, to rostral scale; infralabials (**IL**) = from termination of enlarged scales at the corner of mouth to mental scale; paravertebral tubercles (**PVT**) = number of tubercles between limb insertions counted in a straight line immediately left of vertebral column; total subdigital lamellae beneath 4^th^ toe (**TL4T**); total subdigital lamellae beneath 4^th^ finger (**TL4T**); ventral scales (**VS**) = number of ventral scales across midbody between ventrolateral folds; enlarged femoral scales (**FS**) = number of enlarged scales from each thigh combined as a single metric; total precloaco-femoral pores in males (**PFP**) = total number of continuous pores on the femur and precloacal region.

To eliminate bias stemming from ontogenetic variation (Chan and Grismer 2021), we performed allometric body-size correction using the Thorpe method (Thorpe 1983) implemented in the GroupStruct R package (Chan and Grismer 2022). We then used principal components analysis (PCA) to find the best low-dimensional representation of variation in the data to determine whether morphological variation could form the basis of detectable group structure. The PCA only included closely related species for which published morphometric data are available. These were *Cyrtodactyluspetani* Riyanto, Grismer & Wood, 2015, *C.batucolus* Grismer, Chan, Grismer, Wood & Belabut, 2008, and *C.seribuatensis* Youmans & Grismer, 2006. Only males were included in the analysis because the sample size for females was too low. Using the size-corrected dataset, we performed an ANOVA followed by a TukeyHSD posthoc test to determine whether the means of assessed characters were significantly different among all species pairs. All morphological analyses were performed and visualised in R (R Core Team 2014).

### ﻿Supplementary material

All supplementary material associated with this study can be obtained from the online version of this manuscript and the Figshare repository (https://doi.org/10.6084/m9.figshare.21359970.v1).

## ﻿Results

### ﻿Genetic analyses

**Figure 2.**
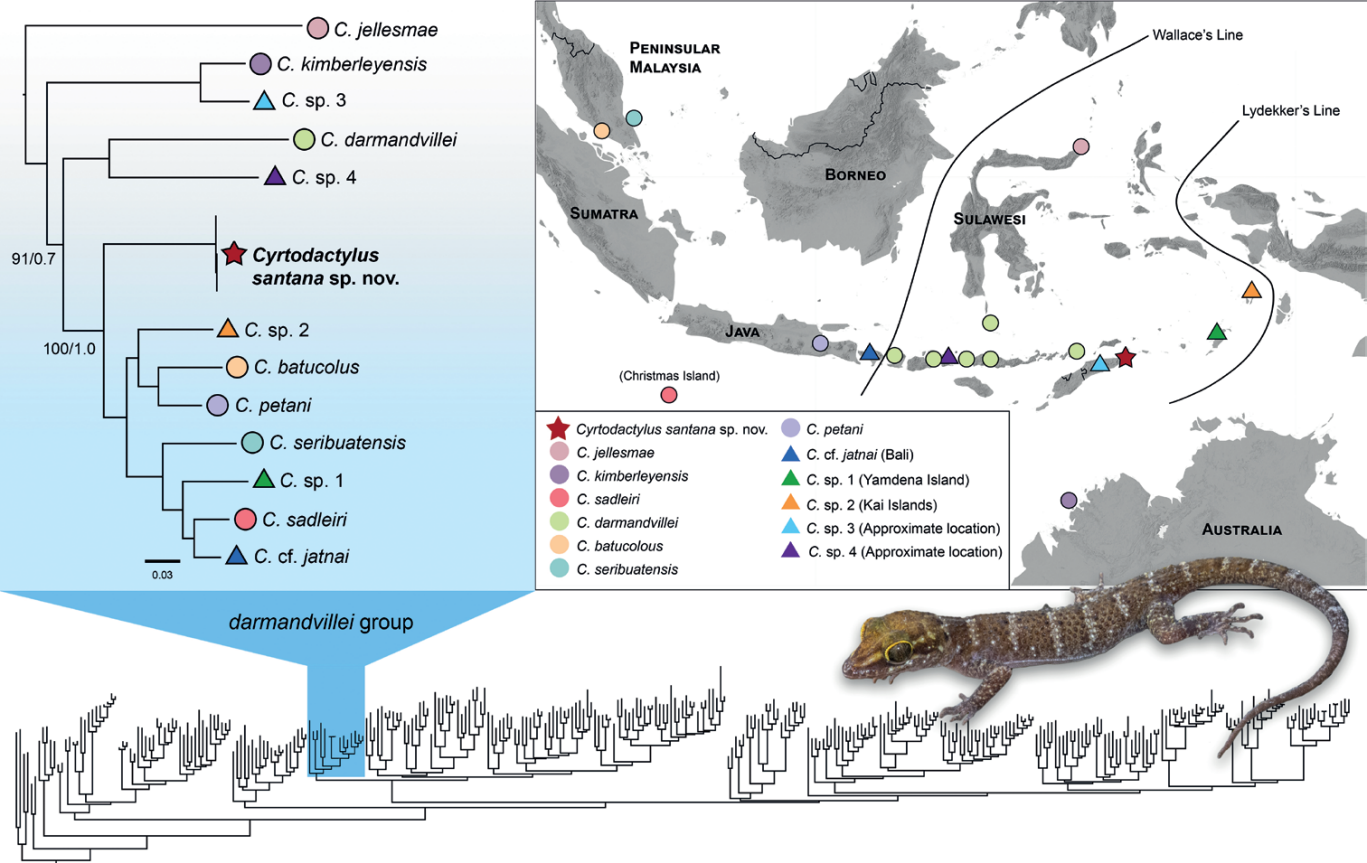
Lower: Genus-wide phylogeny based on all published sequences of described and undescribed lineages of *Cyrtodactylus* (see Suppl. material 1 for the fully annotated phylogeny). The *C.darmandvillei* group is highlighted in blue. Upper left: Maximum-likelihood phylogeny of the *C.darmandvillei* group (Bayesian phylogeny has identical topologies). All nodes are highly supported in the maximum likelihood and Bayesian analysis (UFB/BPP ≥ 99/0.95) except for one node that was moderately supported in the Bayesian analysis (BPP = 0.8). Coloured symbols correspond to the distribution map on the right; Circles = nominal species, Triangles = undescribed/uncertain species, and Star = new species described in this study. Upper right: Distribution of nominal and undescribed lineages in the *C.darmandvillei* group. The specific localities of *C.* sp. 3 (East-Timor) and *C.* sp. 4 (East Nusa Tenggara) are not known, thus, their placements on the map are approximated.

**Figure 3.**
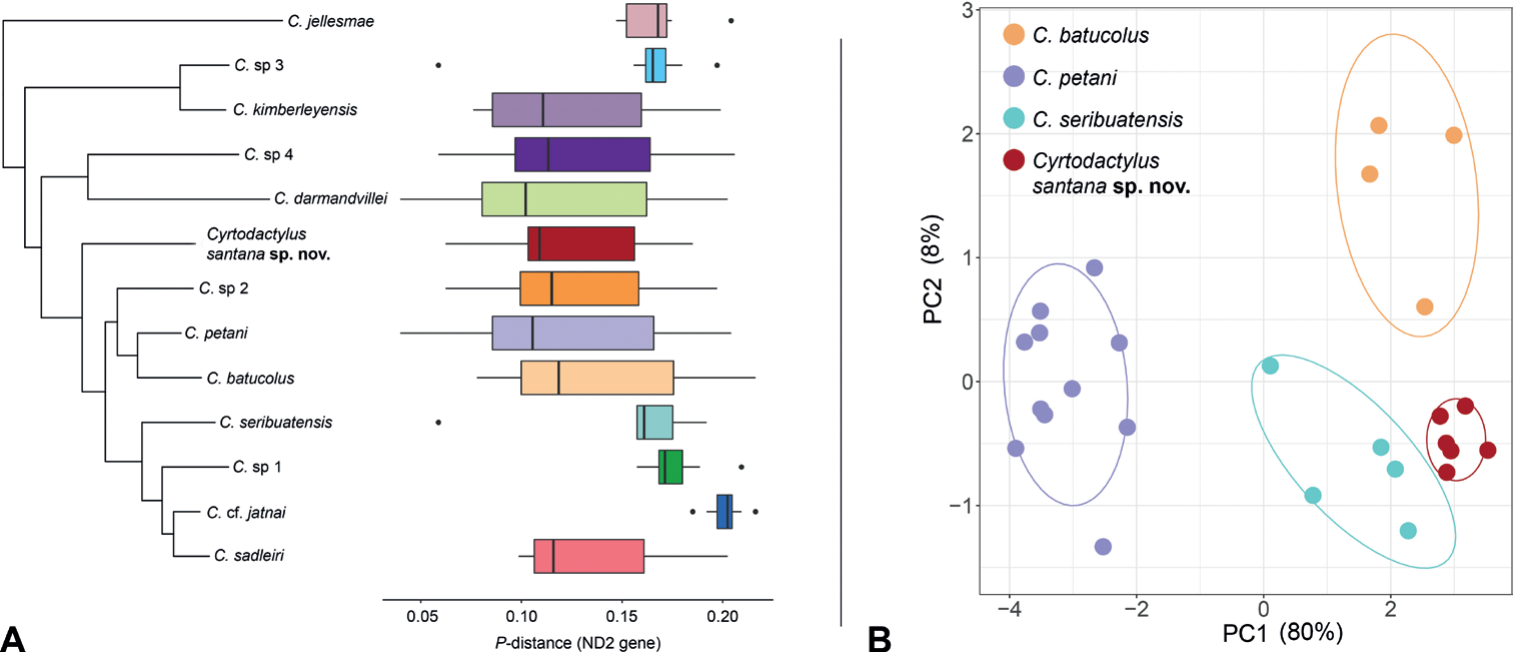
**A** Maximum-likelihood phylogeny of the *Cyrtodactylusdarmandvillei* group with boxplots showing the distribution of pairwise uncorrected *p*-distances (ND2 gene) between each corresponding taxon and all other taxa within the *C.darmandvillei* group **B** plot of PCA scores based on a subset of ten continuous characters.

The final sequence alignment comprised 1566 base pairs, 1297 variable sites, 1141 parsimony informative sites, and 23.2% missing data. The best substitution model scheme for the IQ-TREE analysis was TVM + F + I + G4 for the 1^st^ codon position of ND4 and tRNAs, TIM + F + I + G4 for the 2^nd^ codon position, and GTR + F + I + G4 for the 3^rd^ codon position. The phylogenetic analysis recovered the new population within the *darmandvillei* group (sensu Grismer et al. 2021) with strong support (Ultrafast bootstrap/Bayesian posterior probability, UFB/BPP = 100/1.0; Fig. 2). Within this group, the new population was inferred as the sister lineage to a clade comprising *Cyrtodactylusbatucolus*, *C.petani*, *C.seribuatensis*, *C.sadleiri* Wells & Wellington, 1985, C.cf.jatnai from Bali and two undescribed lineages from Yamdena Island, Indonesia (*C.* sp. 1) and the Kai Islands (*C.* sp. 2) (Fig. 2). Both maximum likelihood and Bayesian phylogenies inferred identical topologies for the *darmandvillei* group with strong support across all taxa except for one branch that received moderate support (UFB/BPP = 91/0.7). Fully annotated and genus-wide phylogenies are included in the Suppl. material 1. Genetic divergence (uncorrected *p*-distance) between the new population and all other taxa in the *darmandvillei* group is high (range = 9.8–20.2%, mean = 13.3%) and consistent with the species-level divergences of other taxa within the *darmandvillei* group (Fig. 3A).

### ﻿Morphological analyses

For the PCA analysis, only PC1 had eigenvalues above 1.0 indicating that most variation (80%) is captured along the first axis (Table 1). Along the first axis (PC1), *Cyrtodactyluspetani* is distinctly separated from the other species and there is a slight separation between *C.seribuatensis* and the new population (Fig. 3B). The second axis (PC2) captured 8% of the variation and further separates *C.batucolus* from *C.seribuatensis* and the new species. PCA loadings for PC1 are relatively consistent (0.27–0.34) indicating that all assessed characters contributed to the overall variation with slightly heavier loadings on ForL, TibL, and HL (Table 1). The fact that *Cyrtodactylussantana* sp. nov., *C.batucolus*, and *C.seribuatensis* overlap along the heavily loaded first PC and all are well-separated from *C.petani* is likely due to the fact that the former are rock-dwellers, and the latter is more of a habitat generalist. Similar results have been observed in *Cyrtodactylus* from Vietnam (Grismer and Grismer 2017) and Peninsular Malaysia (Kaatz et al. 2021).

**Table 1.** Summary statistics and loadings for the PCA analysis. Character abbreviations are defined in Materials and methods.

	PC1	PC2	PC3	PC4	PC5	PC6	PC7	PC8	PC9	PC10
Standard deviation	2.84	0.89	0.57	0.49	0.44	0.41	0.28	0.26	0.22	0.18
Proportion of Variance	0.80	0.08	0.03	0.02	0.02	0.02	0.01	0.01	0.01	0.00
Cumulative Proportion	0.80	0.88	0.92	0.94	0.96	0.98	0.99	0.99	1.00	1.00
Eigenvalue	8.05	0.79	0.33	0.24	0.20	0.17	0.08	0.07	0.05	0.03
SVL	0.31	0.23	0.55	0.28	-0.65	0.11	-0.12	-0.02	0.06	0.09
ForL	0.34	0.13	0.04	-0.23	0.23	-0.37	-0.51	0.04	-0.21	0.57
TibL	0.34	-0.18	-0.14	-0.02	-0.07	-0.11	-0.20	0.78	0.20	-0.36
AG	0.32	0.20	0.45	0.03	0.56	0.03	-0.08	-0.21	-0.07	-0.54
HL	0.34	0.08	-0.05	-0.33	-0.10	0.30	0.44	0.19	-0.66	0.03
HD	0.30	0.33	-0.61	0.43	-0.16	-0.27	-0.02	-0.27	-0.18	-0.22
ED	0.29	-0.51	0.08	0.62	0.29	0.03	0.27	0.05	-0.04	0.33
EE	0.33	-0.02	-0.31	-0.10	0.07	0.74	-0.28	-0.20	0.33	0.12
EN	0.33	0.26	-0.01	-0.26	0.06	-0.26	0.57	-0.04	0.57	0.18
IOD	0.27	-0.64	0.01	-0.34	-0.29	-0.25	-0.05	-0.45	-0.01	-0.21

The ANOVA and TukeyHSD posthoc test showed that the new population is significantly different from *C petani* in all assessed characters; from *C.batucolus* for the characters TibL and IOD; and from *C.seribuatensis* for the characters ForL and EN (Table 2). The morphological analyses of continuous characters indicate that the new population is morphometrically more similar to *C.batucolus* and *C.seribuatensis* than it is to *C.petani*. Comparisons of discrete and meristic characters provide additional distinguishing characters between the new population and other species within the *darmandvillei* group (Table 3).

**Table 2.** Results of the Tukey posthoc test showing the *p*-values for all pairwise comparisons. Values highlighted in green represent *p* < 0.05, whereas those in red represent *p* > 0.05.

	SVL	ForL	TibL	AG	HL	HD	ED	EE	EN	IOD
*petani-batucolus*	0.00	0.00	0.00	0.00	0.00	0.00	0.06	0.00	0.00	0.53
*santana-batucolus*	0.32	0.92	0.04	0.15	0.81	0.78	0.11	0.26	0.69	0.00
*seribuatensis-batucolus*	0.46	0.03	0.94	0.02	0.97	0.02	0.40	0.95	0.00	0.00
*santana-petani*	0.00	0.00	0.00	0.00	0.00	0.00	0.00	0.00	0.00	0.00
*seribuatensis-petani*	0.00	0.00	0.00	0.00	0.00	0.02	0.00	0.00	0.00	0.00
*seribuatensis-santana*	1.00	0.00	0.09	0.58	0.50	0.08	0.86	0.07	0.00	0.48

**Table 3.** Comparisons of discrete and meristic characters among species of the *Cyrtodactylusdarmandvillei* group. NA = not applicable; ? = unknown or not assessable.

	*Cyrtodactylussantana* sp. nov.	*C.batucolus*	*C.darmandvillei*	*C.jellesmae*	*C.kimberleyensis*	*C.petani*	*C.sadleiri*	*C.seribuatensis*
Max SVL	74	75.2	75	63	45	57.2	88	75
Tuberculation moderate to strong	yes	yes	yes	yes	no	yes	yes	yes
Tubercules on forelimbs	yes	yes	yes	yes	no	yes	yes	yes
Tubercules on hindlimbs	yes	yes	yes	yes	no	yes	yes	yes
Tubercules on head and/or occiput	yes	yes	yes	yes	no	yes	yes	yes
Paravertebral tubercles	23–27	30–35	17–20	?	16–18	20–25	22–25	27–35
Proximal subdigital lamellae broad	yes	yes	yes	yes	yes	yes	yes	yes
Subdigital lamellae on 4^th^ toe	15–19	17–19	?	?	16	17–18	19–24	19–22
Ventral scales	42–48	38–42	36–40	40–45	36	30–35	34–42	32–39
Deep precloacal groove	no	no	no	no	no	no	yes	no
Enlarged precloacal scales	yes	yes	?	no	no	yes	yes	yes
Enlarged femoral scales	yes	yes	?	no	no	yes	yes	yes
Precloacal and femoral pores continuous	yes	yes	?	NA	NA	yes	NA	yes
Precloaco-femoral pores	43–45	43–46	?	NA	NA	31–35	NA	42–45
Enlarged median subcaudals	no	no	yes	no	no	no	no	no

### ﻿Systematics

Taken together, the results from our analyses demonstrate that the new population from Timor-Leste is a strongly supported, distinct evolutionary lineage (Fig. 2) that is both genetically (Figs 2, 3A) and morphometrically (Fig. 3B) divergent from its congeners. Therefore, we describe it as a new species below.

#### ﻿ Cyrtodactylus santana sp. nov.

Taxon classificationAnimaliaSquamataGekkonidae

25BA01F1-CB06-5DB5-8220-93BDE80F8B7D

https://zoobank.org/4D481F41-F6F5-4A6E-ABCD-FEFFA8868D2F

**Figure 4.**
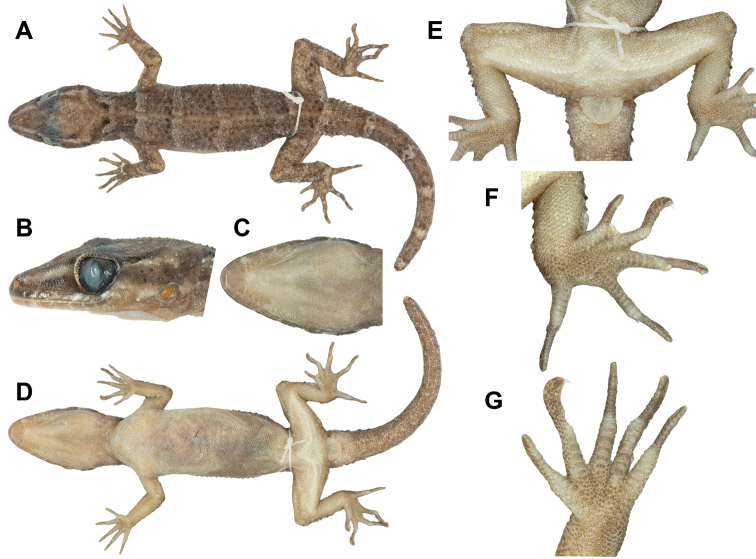
Holotype of *Cyrtodactylussantana* sp. nov. (ZRC 2.7672) **A** dorsal view of entire specimen **B** closeup of side of head **C** closeup of underside of head **D** ventral view of entire specimen **E** ventral view of pelvic region **F** ventral view of right foot **G** ventral view of right hand.

**Figure 5.**
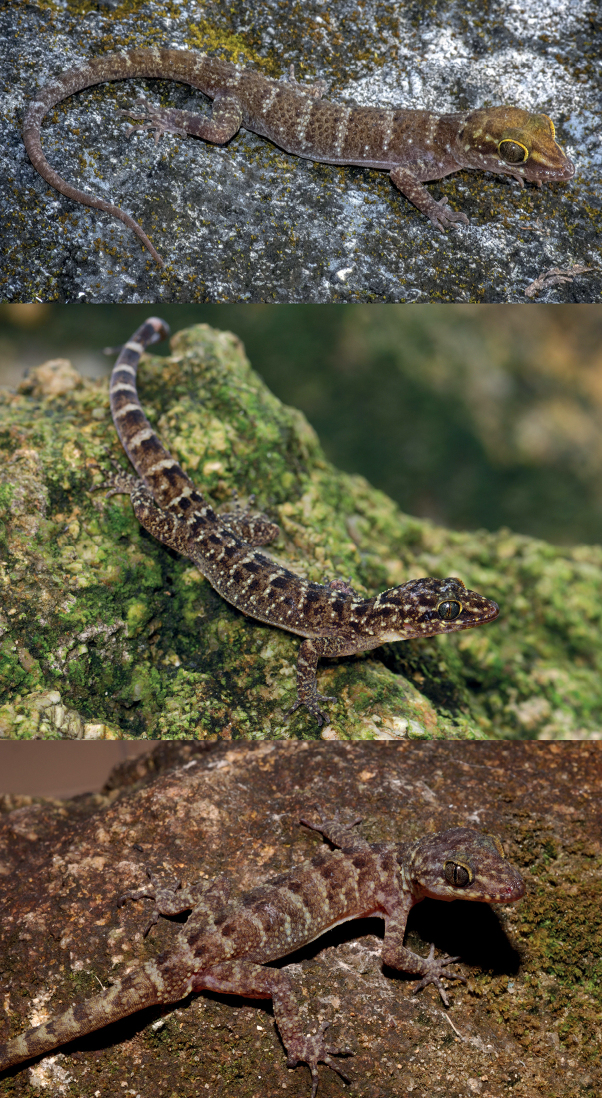
Live images of *Cyrtodactylussantana* sp. nov. paratype female (ZRC 2.7679) (top), *C.batucolus* from Pulau Besar, Malacca, Peninsular Malaysia (middle), and *C.seribuatensis* from Pulau Mentigi, Johor, Peninsular Malaysia (lower).

Figs 4 , 5 (Nino Konis Santana Bent-toed Gecko)

##### Material examined.

***Holotype*.**ZRC 2.7672 (Fig. 4), adult male collected by Chan Kin Onn, Iffah Iesa, Fernando Santana, and Pedro Pinto on 30 August 2022 at 2230 hrs from Napana Wei cave (8.411758°S, 127.293321°E; 152 m a.s.l.) in the northeastern sector of NKS. ***Paratypes*.**ZRC 2.7673–77 (adult males) and ZRC 2.7678–81 (adult females) with the same collection information as the holotype.

##### Diagnosis.

The new species is a distinct evolutionary lineage that is closely related to *C.batucolus*, *C.seribuatensis*, *C.petani*, and *C.sadleiri*. It can be differentiated from other congeners by the following combination of characters: strong dorsal tuberculation present, 23–27 paravertebral tubercles, 15–19 subdigital lamellae on 4^th^ toe, 42–48 ventral scales across midbody, deep precloacal groove absent, enlarged femoral and precloacal scales present, distinct blotches on top of the head absent, dorsal bands faint, whitish, lightly counter-shaded with dark brown.

##### Description of holotype.

Adult male SVL 68.6 mm; head moderate in length (HL/SVL 0.30), wide (HW/HL 0.65), somewhat flattened (HD/HL 0.40), distinct from neck, triangular in dorsal profile; lores weakly inflated, prefrontal region concave, canthus rostralis smoothly rounded; snout elongate (ES/HL 0.43) rounded in dorsal profile; eye large (ED/HL 0.23); ear opening elliptical, moderate in size (EL/HL 0.11), obliquely oriented; eye to ear distance greater than diameter of eye; rostral wider than high, concave, partially divided dorsally, bordered posteriorly by left and right supranasals and smaller medial postrostral (= internasal), bordered laterally by first supralabials; external nares bordered anteriorly by rostral, dorsally by a large, anterior supranasal and small, posterior supranasal, posteriorly by two postnasals, ventrally by first supralabial; 10 (R) 10 (L) squarish supralabials extending to just beyond dorsal inflection of labial margins tapering in size abruptly below midpoint of eye, first supralabial largest; nine (R) and eight (L) infralabials tapering smoothly posteriorly slightly beyond last supralabial posteriorly; scales of rostrum and lores raised, larger than granular scales on top of head and occiput; scales of occiput intermixed with slightly enlarged tubercles; dorsal superciliaries elongate, keeled; mental triangular, bordered laterally by first infralabials and posteriorly by left and right rectangular postmentals which contact medially; one row of slightly enlarged, elongate sublabials extending posteriorly to 6^th^ infralabial; gular scales small, granular, grading posteriorly into slightly larger, flatter, throat scales which grade into larger, flat, smooth, imbricate, pectoral and ventral scales.

Body relatively short (AG/SVL 0.43) with well-defined ventrolateral folds; dorsal scales small, granular, interspersed with moderately sized, conical, semi-regularly arranged, keeled tubercles; tubercles extend from occiput to anterior one-third of tail; tubercles on occiput and nape relatively small, increases in size and density posteriorly; tubercles on pelvic region and hindlimbs largest and densest; approximately 15 longitudinal rows of tubercles at midbody; 27 paravertebral tubercles on body; 44 flat, imbricate, ventral scales between ventrolateral body folds, ventral scales much larger than dorsal scales; precloacal scales large, seven scales across base of precloacal region; precloacal depression weak (Fig. 4E).

Forelimbs moderate in stature, relatively short (ForL/SVL 0.17); granular scales of forearm slightly larger than those of body, interspersed with large, keeled tubercles; palmar scales slightly raised; digits well-developed, inflected at basal, interphalangeal joints; subdigital lamellae transversely expanded throughout its length; digits slightly more narrow distal to inflection; claws well-developed, sheathed by a dorsal and ventral scale; hind limbs more robust than forelimbs, moderate in length (TibL/SVL 0.18), covered dorsally by granular scales interspersed with larger, keeled tubercles and covered anteriorly by flat, slightly larger scales; ventral scales of thigh flat, imbricate, larger than dorsals; ventral tibial scales flat, imbricate; two rows of enlarged, flat, imbricate, femoral scales extend from knee to knee through the precloacal region where they are continuous with enlarged, precloacal scales; posterior row of enlarged femoral scales contains 41 contiguous pore-bearing scales extending from knee to knee forming a V-shape bordering the precloacal depression; postfemoral scales immediately posterior to the row of pore-bearing scales nearly one-half their size, forming an abrupt union on posteroventral margin of thigh; plantar scales low, slightly rounded; digits well-developed, inflected at basal, interphalangeal joints; subdigital lamellae transversely expanded throughout length of digit; digits more narrow distal to joints; 17 subdigital lamellae on right 4^th^ toe, 16 on left; claws well-developed, sheathed by a dorsal and ventral scale.

Tail robust, original, tip broken; dorsal scales at base of tail granular becoming flatter posteriorly; no median row of transversely enlarged, subcaudal scales; subcaudal scales much larger than dorsal caudal scales; one pair of paravertebral and dorsolateral tubercle rows on either side of midline; distance between paravertebral tubercle rows much greater than distance between paravertebral and adjacent dorsolateral rows; caudal tubercles decrease in size posteriorly, extending approximately 40% length of tail; four enlarged, postcloacal tubercles at base of tail on hemipenial swelling; all postcloacal scales flat, imbricate.

##### Colouration in life.

﻿Dorsal ground colour of head yellowish; neck, trunk, limbs, and tail brown; no distinct markings on top of head; pale loreal stripe extend from nostril to eye and continuing as a postorbital stripe that forms a faint forked pattern on occiput; area dorsal and ventral to the loreal and postorbital stripe counter-shaded with dark brown; six pale, faint, thin, irregular bands from nape to base of tail faintly counter-shaded anteriorly and posteriorly with dark brown; dark speckling and faint, cream-coloured blotches on limbs; pale body banding extend onto tail but not encircling tail (Fig. 5). Ventral surfaces of head, body and limbs lightly stippled with grey; subcaudal region darkened with fine mottling; iris greenish brown.

##### Variation.

**Figure 6.**
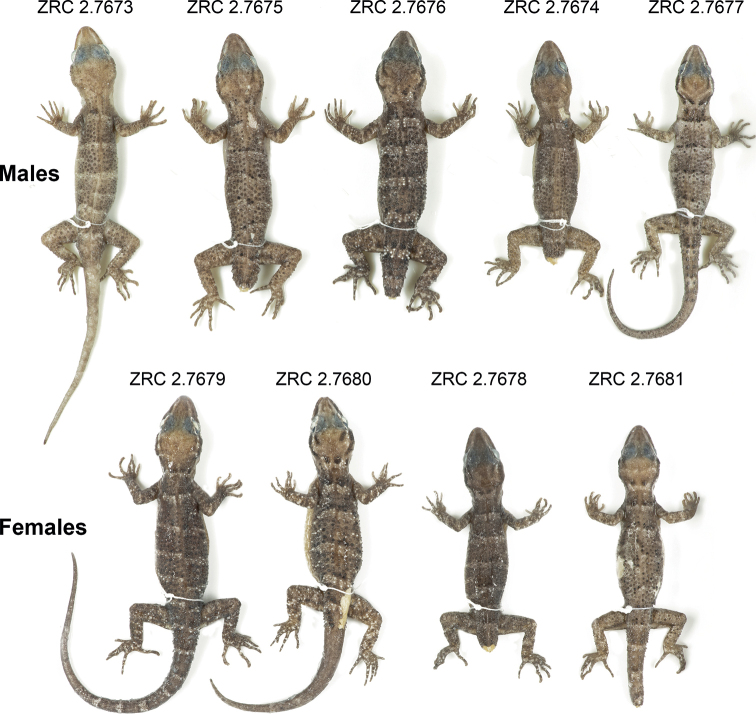
Paratypes of *Cyrtodactylussantana* sp. nov. and their corresponding voucher numbers.

ZRC 2.7674–76, ZRC 2.7678, and ZRC 2.7680–81 have broken tails. Some specimens have more distinct dorsal markings than others (Fig. 6). The level of yellowness of the head also varies and does not appear to be a sexually dimorphic character. Meristic differences are listed in Table 4.

**Table 4.** Raw morphological data for the type series. Character abbreviations are defined in Materials and methods.

	ZRC 2.7672	ZRC 2.7673	ZRC 2.7674	ZRC 2.7675	ZRC 2.7676	ZRC 2.7677	ZRC 2.7678	ZRC 2.7679	ZRC 2.7680	ZRC 2.7681
Type	Holotype	Paratype	Paratype	Paratype	Paratype	Paratype	Paratype	Paratype	Paratype	Paratype
Sex	male	male	male	male	male	male	female	female	female	female
SVL	68.6	70	64.6	74	70.6	59.1	62.2	70	64.2	60.6
AG	29.6	28.7	25.8	32.3	28	24.3	27.5	31.6	27.5	27.2
HumL	7.7	8.3	7.8	8.8	9	6.9	7.6	8.6	7.9	6.7
ForL	11.5	11.2	10	12	11.8	10	9.8	10.8	10	9.5
FemL	17.6	17	14.8	17.1	16.3	14.4	15	17.2	16.6	14
TibL	12.1	12	11.8	13.7	13.5	11	11.8	12.9	12	11.6
HL	20.5	20.7	19.2	22.7	21.5	17.1	18.4	20.7	19.2	17.9
HW	13.5	14	12.4	14.7	14.9	11.9	11.6	13.6	13.3	12
HD	8.2	9.3	8	9.1	9.8	7.3	7.4	8.6	8.5	7.4
ED	4.8	5.2	4.2	4.8	5.1	4.2	4.3	5.2	5.2	4
EE	5.8	5.7	5.9	6.4	6.3	5	5.4	5.7	5.3	4.7
ES	8.9	8.7	8	9.8	8.8	7.8	7.5	9.2	8.2	7.7
EN	6.4	6.7	6.5	7.1	6.8	5.7	6	6.7	6.2	5.7
IO	6.2	6.1	5.7	6.8	6.3	5.2	5.4	6	6	5.6
EL	2.3	2.2	1.5	2.3	1.7	1.4	1.9	1.8	2	1.2
IN	2.4	2.8	2.2	2.3	2.3	2	2	2.4	1.9	1.8
SL (R/L)	10/10	10/11	11/11	11/12	12/11	10/10	11/11	11/11	10/11	12/11
IL (R/L)	9/8	8/8	9/9	9/9	10/9	9/9	10/9	9/9	9/9	9/9
PVT	27	25	27	27	25	23	26	27	24	24
TL4T	17	15	16	18	19	15	15	15	15	16
TL4F	17	17	17	17	18	15	18	18	18	18
VS	44	45	48	42	48	45	47	47	42	42
FS	28	28	28	26	27	25	27	27	26	26
PFP	44	45	43	45	45	?	NA	NA	NA	NA

##### Comparisons.

Due to the large number of *Cyrtodactylus* species, we restrict our comparison to species within the *darmandvillei* group. The new species differs from *C.batucolus* by having fewer paravertebral tubercles (23–27 vs. 30–35), more ventral scales (42–48 vs. 38–42), lacking distinct blotches on top of a yellowish head, lacking dark paravertebral dorsal blotches on the body and tail, and having less distinct but well-defined pale-coloured dorsal bands (Fig. 5). From *C.darmandvillei* Weber, 1890, it differs by having more paravertebral tubercles (23–27 vs. 17–20), more ventral scales (42–48 vs. 36–40), and lacking enlarged median subcaudals. From *C.jellesmae* Boulenger, 1897 it differs by being larger in size (max SVL 74 mm vs. 63 mm) and having as opposed to lacking enlarged femoral and precloacal scales. From *C.kimberleyensis* Bauer & Doughty, 2012, it differs by being larger in size (max SVL 74 mm vs. 45 mm), having moderate to strong dorsal tubercles (vs. weak to absent), more paravertebral tubercles (23–27 vs. 16–18), more ventral scales (42–48 vs. 36), and having as opposed to lacking enlarged femoral and precloacal scales. From *C.petani*, it differs by being larger in size (max SVL 74 mm vs. 57.2 mm), having more ventral scales (42–48 vs. 30–35), and more precloaco-femoral pores in males (43–45 vs. 31–35). From *C.sadleiri*, it differs by being smaller in size (max SVL 74 mm vs. 88 mm), having less subdigital lamellae on 4^th^ toe (15–19 vs. 19–24), more ventral scales (42–48 vs. 34–42), and lacking a deep precloacal groove. From *C.seribuatensis*, it differs by having fewer subdigital lamellae on 4^th^ toe (15–19 vs. 19–22), more ventral scales (42–48 vs. 32–39), and lacking distinct blotches on top of a yellowish head, lacking dark paravertebral dorsal blotches on the body and tail, and having less distinct but well-defined pale-coloured dorsal bands (Fig. 5).

##### Distribution.

*Cyrtodactylussantana* sp. nov. occurs in Lene Hara and Napana Wei caves within NKS. The nearest village is Tutuala in the municipality of Lautém. The larger distribution of this species is not yet known but it likely occurs in other limestone caves within NKS. There is a report of a similar-looking and unidentified *Cyrtodactylus* on Ataúro island (Kaiser et al. 2013: fig. 3B). However, the specific identity of the Ataúro *Cyrtodactylus* cannot be ascertained at this point due to the lack of comparative material. As such, we consider the distribution of *Cyrtodactylussantana* sp. nov. to be restricted to NKS until new data suggest otherwise (see Discussion for more information about the *Cyrtodactylus* from Ataúro).

##### Natural history.

**Figure 7.**
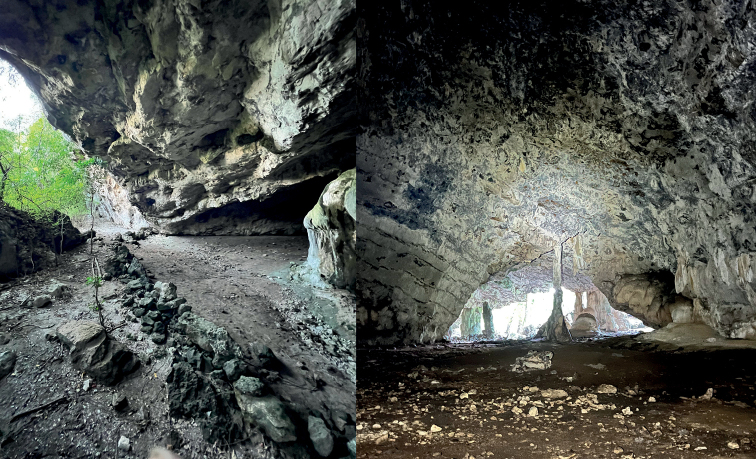
Type locality of *Cyrtodactylussantana* sp. nov. at Lene Hara cava as viewed from the exterior (left) and interior (right). Photographs by Tan Heok Hui.

Lizards were considerably more abundant in Napana Wei cave compared to Lene Hara cave (Fig. 7) even though the two caves are adjacent to each other and are less than 500 m apart but not connected by contiguous limestone. This disparity could be associated with the differences in the geomorphology of both caves. Lene Hara cave is cavernous and dome-like with a high ceiling (Fig. 7), whereas Napana Wei is low and narrow. In Lene Hara, a small number of lizards were observed under small rocks and on columnar formations but in Napana Wei, lizards were found in abundance on the underside and exterior surface of the cave wall. No lizards were observed on surrounding vegetation, suggesting that they could be limestone specialists. The caves are located less than 1 km from the coast. *Cyrtodactylussantana* sp. nov. is nocturnal and is found in sympatry with *Gehyra* sp.

##### Etymology.

Nino Konis Santana was a freedom fighter who led the Falintil militia against the Indonesian occupation of Timor-Leste. He was not only a fearless leader of the armed wing of the Resistance but also played a key role in peace initiatives, earning him a reputation as a peacemaker, diplomat, and statesman. The Nino Konis Santana National Park was named in honor of this national hero who was born in the suco (village in Tetum) of Tutuala, located within the boundaries of the park. The specific epithet *santana* is used as a noun in apposition referring to Nino Konis Santana National Park, which is the type locality of the new species.

## ﻿Discussion

Members of the *darmandvillei* group comprise lineages from Wallacea (*Cyrtodactylussantana* sp. nov., *C.jellesmae*, *C.darmandvillei*, *C.* sp. 1, *C.* sp. 2, *C.* sp. 3, and *C.* sp. 4), Sundaland (*C.batucolus*, *C.petani*, *C.seribuatensis*, *C.sadleiri*, C.cf.jatnai), and an island off the northern tip of Western Australia (*C.kimberleyensis*; Fig. 2). However, the most recent common ancestors of the non-Wallacean lineages are younger than the Wallacean lineages suggesting that the ancestor of the *darmandvillei* group likely originated in Wallacea with more recent dispersals into Sundaland and Australia (Grismer et al. 2022). It is interesting to note that members of the *darmandvillei* group are absent in the greater Sunda islands of Borneo and Sumatra but are present on islands off the eastern and western coast of southern Peninsular Malaysia (*C.batucolus* and *C.seribuatensis*). Stranger still are the phylogenetic placements of *C.batucolus* and *C.seribuatensis* from Peninsular Malaysia, which are more closely related to lineages from Java and the Moluccas, respectively, than they are to each other (Fig. 2). This anomalous pattern could be attributed to missing taxa that have yet to be discovered or taxa that lack genetic representation, particularly from Sulawesi and the Moluccas. There are numerous species of *Cyrtodactylus* described from Wallacea that have not yet been sequenced including *C.batik* Iskandar, Rachmansah & Umilaela, 2011, *C.celatus* Kathriner, Bauer, O’Shea, Sanchez & Kaiser, 2014, *C.fumosus* (Müller, 1895), *C.halmahericus* (Mertens, 1929), *C.deveti* (Brongersma 1948), *C.hitchi* (Riyanto et al. 2016), *C.nuaulu* (Oliver et al. 2009), *C.tahuna* (Riyanto et al. 2018b), *C.tanahjampea* Riyanto, Kurniati & Engilis, 2018, *C.tambora* Riyanto, Mulyadi, McGuire, Kusrini, Febylasmia, Basyir & Kaiser, 2017, *C.wetariensis* (Dunn 1927), and *C.wallacei* Hayden, Brown, Gillespie, Setiadi, Linkem, Iskandar, Umilaela, Bickford, Riyanto, Mumpuni & McGuire, 2008. Although most Wallacean taxa belong to the *darmandvillei* group, there is one known exception, *C.papeda* Riyanto, Faz, Amarasinghe, Munir, Fitriana, Hamidy, Kusrini & Oliver, 2022 from Obi Island in the Moluccas that is part of the *marmoratus* group. This is a relatively small group comprising species that are distributed in Java, Sumatra, the Moluccas, and New Guinea (Grismer et al. 2021); and are notably absent in the intervening regions of the Lesser Sunda Islands and Sulawesi. Nevertheless, we expect that most Wallacean taxa will fall within the *darmandvillei* group barring a few exceptions. We anticipate that the eventual inclusion of sequences from missing taxa in a comprehensive phylogenetic analysis will reveal new insights and improve our understanding of the biogeography and evolutionary history of *Cyrtodactylus* in the Southeast Asian region and beyond.

Our phylogenetic analysis also included several lineages of uncertain identities. Riyanto et al. (2015) published a phylogeny that included two lineages of *Cyrtodactylus* from Bali, one of which we consider to be conspecific with *C.seribuatensis* based on low genetic differentiation. Subsequently, *Cyrtodactylusjatnai* Amarasinghe, Riyanto, Mumpuni & Grismer, 2020 was described from Bali solely based on morphology and was demonstrated to be distinct from *C.seribuatensis*. We hypothesise that the unidentified sequence from Bali (GenBank KU232624) could represent *C.jatnai* and therefore we refer to that sequence as C.cf.jatnai pending confirmation from additional data. There is also an unidentified lineage from Timor-Leste (*C.* sp. 3) that is more closely related to *C.kimberleyensis* from Australia than it is to *Cyrtodactylussantana* sp. nov. indicating that *Cyrtodactylus* on Timor-Leste is not monophyletic. Based on the general colour pattern and small size of *C.kimberleyensis* (Bauer and Doughty 2012), we believe that *C.* sp. 3 could be one of the unidentified species shown in O’Shea et al. (2015), all of which are relatively small in size and bear broad morphological resemblance to *C.kimberleyensis*.

Based on currently available data, *Cyrtodactylussantana* sp. nov. is a nocturnal species occurring in limestone caves in the lowland tropical forest of NKS. We did not observe any lizards on the vegetation surrounding the caves. However, our observations are based on one night’s sampling effort and our supposition that this species occurs exclusively on limestone could be overturned with more extensive and intensive surveys. There is also a report of a similar-looking and unidentified *Cyrtodactylus* on Ataúro Island that is referred to as *C.* sp. ‘Ataúro coast’ in O’Shea et al. (2015). In that report, the authors noted that the lizard was superficially similar to *C.darmandvillei* and was found in a variety of habitats including a limestone cliff, coconut groves, rock piles, and a tropical dry forest. Unfortunately, due to the lack of comparative material, the specific identity of *C.* sp. ‘Ataúro coast’ cannot be determined at this point. We hypothesise that the population from Ataúro could either be conspecific with *Cyrtodactylussantana* sp. nov. or represent a closely related but distinct lineage that is yet to be described. It is worth noting that *Cyrtodactylussantana* sp. nov. is the first *Cyrtodactylus* with a specific identity in Timor-Leste as all previous reports of the genus were not identified at the species level (Kaiser et al. 2011, 2013; O’Shea et al. 2015), a clear indication that the diversity of *Cyrtodactylus* in Timor-Leste is still underestimated and poorly understood.

## Supplementary Material

XML Treatment for Cyrtodactylus santana

## Supplementary materials

Supplementary material 1 Supplementary information Data type zip file Explanation note Maximum Clade Credibility Bayesian tree inferred using BEAST. Maximum Likelihood consensus tree inferred using IQ-TREE. Raw (not size corrected) measurements in mm used in the morphological analyses. Information on all sequences used in this study and their associated GenBank accession numbers (Suppl. material 1: table S1). https://binary.pensoft.net/file/794109This dataset is made available under the Open Database License (http://opendatacommons.org/licenses/odbl/1.0/). The Open Database License (ODbL) is a license agreement intended to allow users to freely share, modify, and use this Dataset while maintaining this same freedom for others, provided that the original source and author(s) are credited.Kin Onn Chan, L. Lee Grismer, Fernando Santana, Pedro Pinto, Frances W. Loke, Nathan Conaboy
